# Characterization of PbPga1, an Antigenic GPI-Protein in the Pathogenic Fungus *Paracoccidioides brasiliensis*

**DOI:** 10.1371/journal.pone.0044792

**Published:** 2012-09-14

**Authors:** Clarissa X. R. Valim, Luiz Roberto Basso, Fausto B. dos Reis Almeida, Thaila Fernanda Reis, André Ricardo Lima Damásio, Luisa Karla Arruda, Roberto Martinez, Maria Cristina Roque-Barreira, Constance Oliver, Maria Célia Jamur, Paulo Sergio Rodrigues Coelho

**Affiliations:** 1 Departamento de Biologia Celular e Molecular e Biagentes Patogênicos, Faculdade de Medicina de Ribeirão Preto, São Paulo, Brazil; 2 Departamento de Bioquímica e Imunologia, Faculdade de Medicina de Ribeirão Preto, São Paulo, Brazil; 3 Departamento de Clínica Médica, Faculdade de Medicina de Ribeirão Preto, São Paulo, Brazil; Universidade de Sao Paulo, Brazil

## Abstract

*Paracoccidioides brasiliensis* is the etiologic agent of paracoccidioidomycosis (PCM), one of the most prevalent mycosis in Latin America. *P. brasiliensis* cell wall components interact with host cells and influence the pathogenesis of PCM. Cell wall components, such as glycosylphosphatidylinositol (GPI)-proteins play a critical role in cell adhesion and host tissue invasion. Although the importance of GPI-proteins in the pathogenesis of other medically important fungi is recognized, little is known about their function in *P. brasiliensis* cells and PCM pathogenesis. We cloned the *PbPga1* gene that codifies for a predicted GPI-anchored glycoprotein from the dimorphic pathogenic fungus *P. brasiliensis*. *PbPga1* is conserved in Eurotiomycetes fungi and encodes for a protein with potential glycosylation sites in a serine/threonine-rich region, a signal peptide and a putative glycosylphosphatidylinositol attachment signal sequence. Specific chicken anti-rPbPga1 antibody localized PbPga1 on the yeast cell surface at the septum between the mother cell and the bud with stronger staining of the bud. The exposure of murine peritoneal macrophages to rPbPga1 induces TNF-α release and nitric oxide (NO) production by macrophages. Furthermore, the presence of *O*-glycosylation sites was demonstrated by β-elimination under ammonium hydroxide treatment of rPbPga1. Finally, sera from PCM patients recognized rPbPga1 by Western blotting indicating the presence of specific antibodies against rPbPga1. In conclusion, our findings suggest that the *PbPga1*gene codifies for a cell surface glycoprotein, probably attached to a GPI-anchor, which may play a role in *P. brasiliensis* cell wall morphogenesis and infection. The induction of inflammatory mediators released by rPbPga1 and the reactivity of PCM patient sera toward rPbPga1 imply that the protein favors the innate mechanisms of defense and induces humoral immunity during *P. brasiliensis* infection.

## Introduction

*Paracoccidioides brasiliensis* is a thermal dimorphic fungus and the etiological agent of paracoccidioidomycosis (PCM), the most prevalent systemic mycosis in Latin America [1] Infection occurs primarily in the lungs through inhalation of conidia or hyphal particles that switch to yeast form at the human body temperature [2]. *P. brasiliensis* may remain latent for long periods and the progression to disease depends on the host-pathogen interplay [3]. PCM may present multiple clinical manifestations, ranging from localized to systemic mycosis, which is disseminated via the bloodstream and/or the lymphatic system [4], [5].

The fungal cell wall is a dynamic and highly regulated structure where several molecules are important for cell wall synthesis and maintenance and in the interaction with host tissues. Extensive changes in the composition and arrangement of the cell wall may occur during fungal morphogenesis that is triggered by environmental signals [6]–[8]. Cell wall glycosylphosphatidylinositol (GPI)-anchored proteins have been extensively studied in *Candida albicans* and *Saccharomyces cerevisiae* [9], [10]. In these organisms, GPI-proteins are involved in cell wall integrity, as well as in pathogenic processes such as adhesion and degradation of host tissue and immune response [9], [11]–[13]. The GPI-proteins frequently display a common structural organization that allow their identification using *in silico* approaches based on genome sequence [14], [15]. In general, GPI-proteins have an N-terminal signal peptide for translocation across the membrane of the endoplasmic reticulum and a C-terminal consensus sequence for GPI attachment. In addition, many of these proteins, adhesins e.g., have a central Ser-Thr (serine/threonine) rich *O*-glycosylated domain [16].

The GPI-proteins from *Aspergillus* species have been the most studied of the mycelial fungi. Bioinformatic approaches have identified more than one hundred GPI-protein candidates in the genomes of *A. fumigatus*, *A. nidulans* and *A. oryzae* [15], [17], [18]. Studies in *Aspergillus* and *Neurospora* have revealed the role of GPI-proteins in many biological processes such as hyphal cell wall assembly, morphogenesis, germination, hyphal growth, adhesion, immune response and virulence [15], [18]–[30]. Although identified in mycelial fungi, it is controversial whether these GPI-proteins share the same overall pattern of amino acid composition (e.g. Ser-Thr content) and structural modularity that characterizes the GPI-proteins described in Saccharomycetes [18]. Furthermore, it is still unclear whether the whole set of GPI-proteins from mycelial fungi may be grouped into similar or additional functional classes such as adhesins or biosynthetic enzymes as observed for *S. cerevisiae*. Previous studies have suggested that some GPI-proteins from mycelial fungi may play distinct functions in cell wall integrity and pathogenesis. For example, the β(1–3) glucanosyltransferase encoded by *Gel4* gene from *A. fumigatus* [30] is the only characterized member of this family known to be essential in fungi. Therefore, the identification and characterization of new GPI-proteins in fungi may allow a better understanding of their cell wall organization and the pathogenic processes. In addition GPI-proteins may be targets for new antifungal agents or be used as vaccines.

In *P. brasiliensis*, a group of 20 predicted GPI-proteins was identified based on an EST database using an *in silico* analysis [31], [32]. Despite the efforts to identify GPI-proteins, the majority of predicted GPI-proteins from *Paracoccidioides* genome is uncharacterized. In this context, the present study was undertaken to identify and characterize a novel predicted GPI-protein from *P. brasiliensis*, PbPga1, on the surface of yeast cells. PbPga1is up-regulated in the pathogenic yeast form, the recombinant rPbPga1 was able to activate peritoneal macrophages, and was recognized by sera from PCM patients.

## Results

### Identification of PbPga1 in *P. brasiliensis* strain Pb18

**Figure 1:**
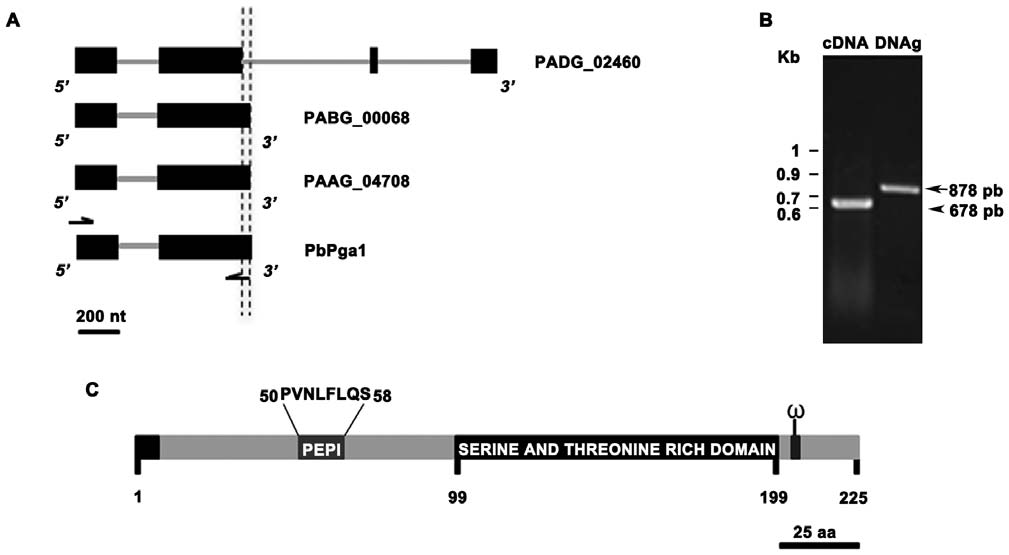
Identification of *PbPga1* from *P. brasiliensis*. (A) Exon/intron structure of *PbPga1* and its orthologs from *P. lutzii* strain Pb01 (PAAG_04708), *P. brasiliensis* strain Pb03 (PABG_00068) and the ORF PADG_02460 from *P. brasiliensis* strain Pb18, as predicted by the Fungal Genome Initiative of the Broad Institute. Exons are represented by boxes and introns are represented by lines. The vertical dashed lines indicate the 3′ boundaries in the second intron of PADG_02460 and in the reannotated *PbPga1*. (B) GelRed stained agarose gel of PCR products after amplification with primers BamHI_PbPga1-F and the primer HindIII_PbPga1-R2 using genomic DNA (gDNA) or cDNA as templates. kb: Kilobase. (C) Predicted PbPga1 has a total size of 225 amino acids (aa). PbPga1 presents a signal peptide at its N-terminal region (aa 1–22), followed by a non-glycosylated region (aa 23–98). In order to produce an antibody against the non-glycosylated region of PbPga1 a peptide of 8 amino acids (Pep I) was designed starting at aa 50 ending at aa 58. An *O-*glycosylated serine and threonine rich domain starts at position aa 99 ending at aa 199. An omega-site is present in the C-terminal region (ω).

In order to discover non-characterized GPI-proteins, we performed a tBLASTp search using the whole set of the Expressed Sequence Tags (EST) from *P. brasiliensis* (Marques et al., 2004) as queries against the genomes of *P. brasiliensis* strains (Pb03 and Pb18) and *P. lutzii* strain (Pb01) at the Broad Institute. We identified a cDNA clone homologous to three open reading frames (ORFs) (PAAG_04708, PABG_00068 and PADG_02460) that contain GPI-protein signals, except for PADG_02460 C-terminus (Figures 1A and S1). Manual reannotation of PADG_02460 exon/intron structure revealed a 678 bp ORF that contains a potential C-terminal GPI signal (Figure 1 and S1). The reannotation of PADG_02460 ORF was confirmed by DNA sequencing of PCR products using genomic DNA or cDNA as templates (Figure 1B). This ORF was named *P. brasiliensis* Predicted GPI anchored protein one (*PbPga1*). Quantitative Real Time PCR (qRT-PCR) analysis showed that *PbPga1* RNA levels are 7-fold higher in yeast than in hyphal cells (data not shown). The predicted protein PbPga1 presents the modular domains characteristic of fungal GPI-proteins. At the N-terminal region it presents a signal peptide sequence required for transferring the protein into the endoplasmic reticulum (ER), followed by a small non-glycosylated region, a serine and threonine *O-glyc*osylated rich domain, and an omega-site (ω-site, GPI-anchored site) in its C-terminal portion (Figure 1C).

### *P. brasiliensis* PbPga1 is restricted to Eurotiomycetes

We searched fungal genome databases at NCBI and Broad Institute using the PbPga1 sequence as a query to identify PbPga1 orthologs (Table 1). A multiple alignment of PbPga1 sequence and its orthologs generated a neighbor-joining phylogenetic tree (Figure S2). PbPga1 showed the highest homology to the proteins encoded by PABG_00068 and PAAG_04708 (90% and 79%) from *P. brasiliensis* strain Pb03 and *P. lutzii* strain Pb01, respectively. Interestingly, PbPga1 orthologs were found exclusively within the Eurotiomycetes class. The phylogenetic analysis reveals that PbPga1 and its orthologs follow the phylogenetic parenthood of Eurotiales and Onygenales orders (Figure S2). The sequences of PbPga1 and its orthologs were investigated for conserved domains found in GPI-proteins (Table 1). All the sequences analyzed have secretion signal at the N-terminus, serine/threonine rich regions and a predicted ω-site at C-terminus (Table 1).

**Table 1:** *PbPga1* orthologs in different fungi.

NAME	GI#	Organism	Length (a.a.)	M.W. (kDa)	Score	ω site	SignalP	S/T (%)
*PbPga1*	JN967755	*Paracoccidioides brasiliensis* (Pb18)	225	23.2	100	+	+	36.9
PADG_02460	226290934	*Paracoccidioides brasiliensis* (Pb18)	267	27.8	94.0	−	+	35.2
PABG_00068	225679221	*Paracoccidioides brasiliensis* (Pb03)	225	23.1	90.0	+	+	38.2
PAAG_04708	295665256	*Paracoccidioides lutzii* (Pb01)	225	23	79.0	+	+	37.3
HCAG_02716.3	154283499	*Histoplasma capsulatum*	211	21.5	45.0	+	+	32.7
BDBG_04225	261197908	*Blastomyces. dermatitidis*	217	22.2	44.0	+	+	34.1
MGYG_05073	315046212	*Microsporum gypseum*	230	23.4	39.0	+	+	37.8
CIMG_02211	119195673	*Coccidioides immitis*	222	22.1	37.0	+	+	29.7
CPC735_038790	303321532	*Coccidioides posadasii*	222	22.1	37.0	+	+	30.2
MCYG_07683	238845202	*Microsporum canis*	232	23.2	36.0	+	+	38.3
ARB_06477	302506795	*Arthroderma benhamiae*	231	23.8	36.0	+	+	35.0
TERG_01967	327305107	*Trichophyton rubrum*	233	23.9	35.0	+	+	36.0
TEQG_00169.2	326477105	*Trichophyton equinum*	234	24.0	34.0	+	+	38.0
AFL2G_12303	238508443	*Aspergillus flavus*	248	24.7	34.0	+	+	42.2
ATEG_00050	114196996	*Aspergillus terreus*	239	23.9	34.0	+	+	36.8
PMAA_059950	210068124	*Penicillium marneffei*	224	22.7	34.0	+	+	37.5
AFU8G04370	70983434	*Aspergillus fumigatus*	221	22.5	33.0	+	+	39.3
TSTA_011620	218716632	*Talaromyces stipitatus*	245	24.1	32.0	+	+	42.4
NFIA_097100	119410140	*Neosartorya fischeri*	219	22.3	31.0	+	+	38.8
ANI_01449	67521984	*Aspergillus nidulans*	232	23.9	30.0	+	+	40.5
ACLA_057720	119404754	*Aspergillus clavatus*	228	22.9	30.0	+	+	40.8

Score = Percentage of identity relative to *PbPga1* calculated by CLUSTALW2 program.

GI# = Genbank identification number.

ω site = Omega site predicted by FragAnchor and GPI-SOM programs.

SignalP = Signal peptide cleavage site predicted by SignalP3.0 program.

a.a. = Amino acid.

M.W. = Molecular weight predicted by Bioedit Sequence Alignment Editor program.

### Expression of rPbPga1 in *Pichia pastoris*

**Figure 2:**
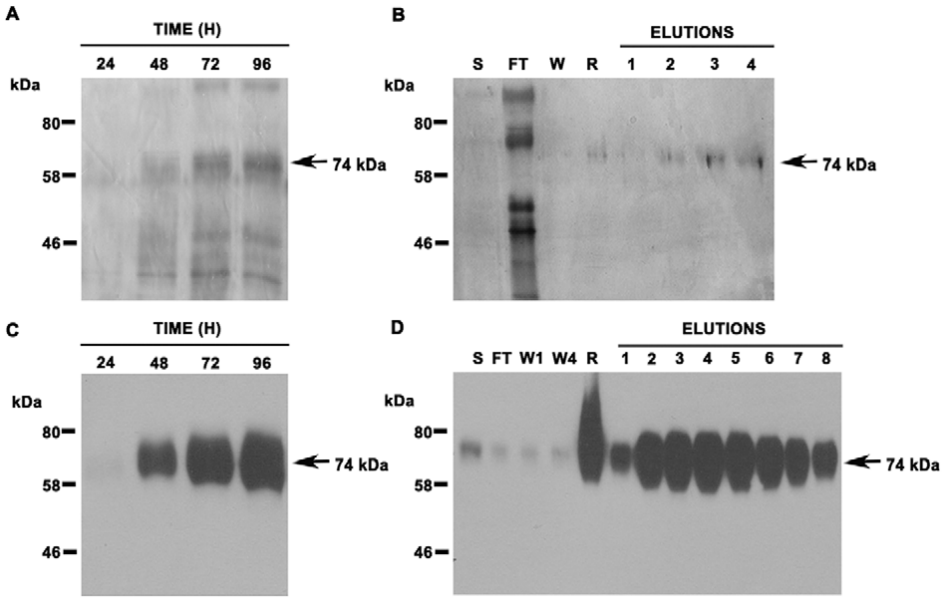
Purification of rPbPga1 expressed in *P. pastoris*. Supernatant samples from induced *Pp*CV1 cells were analyzed by (A) silver staining of SDS-PAGE gels and by (C) Western blot using an anti-His antibody (1∶1000). The supernatant (S) from induced *Pp*CV1 cells was subjected to a nickel column and eluted samples were analyzed (B) by silver stained SDS PAGE gel and (D) Western blot using anti-His antibody (1∶1000). A polypeptide of 74 kDa corresponding to rPbPga1 was detected in all samples (arrows). Flow through sample (FT). Unbound washed sample (W1 and W4). Resin bound rPbPga1 (R).

The PbPga1 full length protein was expressed in a eukaryotic system (*Pichia pastoris*) which allows for post-translational modifications such as glycosylation. Initially, *P. pastoris* cells were transformed with the native *PbPga1* ORF but did not yield a polypeptide in induced cells (data not shown). Considering that the lack of PbPga1 expression might be caused by codon bias, a synthetic *PbPga1* gene (*rPbPga1)* was designed with optimized *P. pastoris* codons. The *rPbPga1* ORF lacks the GPI signal at the C-terminus and a His-tag coding sequence was fused in frame to *PbPga1* ORF. *P. pastoris* cells transformed with *rPbPga1* (PpCV1) were induced by methanol during different time points and the supernatants were analyzed by Western blotting using anti-His antibody. A polypeptide of 74 kDa was identified after 48 hours of induction (Figure 2A and C). After 96 hours of induction, the culture supernatant was loaded onto Ni-nitrilotriacetic acid (NTA) column (Figure 2B and D). The identity of rPbPga1 was confirmed by mass spectrometry of the eluted fraction (Figure S3).

### Characterization of IgY anti-rPbPga1

**Figure 3:**
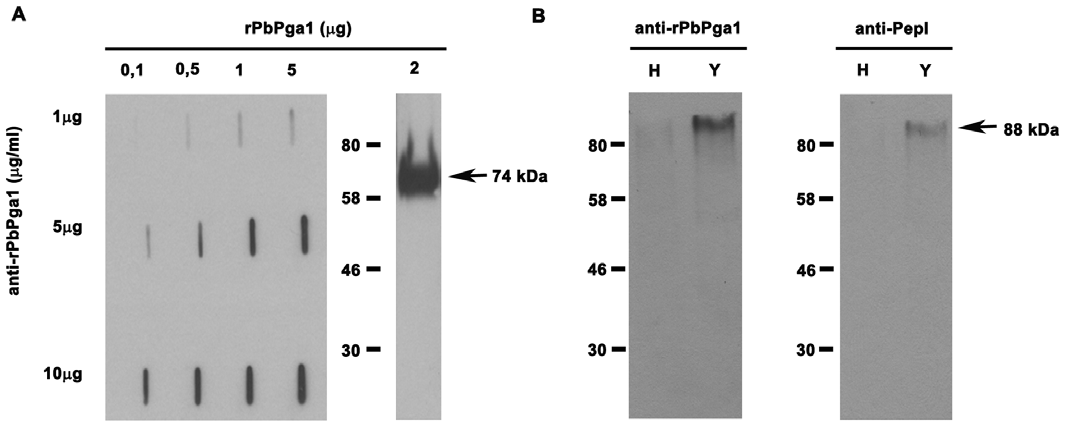
Characterization of IgY anti-rPbPga1. (A) Anti-rPbPga1 was titered by slot blot and tested for specificity. Membranes containing purified rPbPga1 (0.1 µg, 0.5 µg, 1 µg and 5 µg) were incubated with anti-rPbPga1 at different concentrations (1 µg, 5 µg and 10 µg). When rPbPga1 (2 µg) was subjected to Western blotting using anti-rPbPga1 (5 µg), a single 74 kDa polypeptide (arrow) was detected. (B) Western blot analysis of the total protein extract (50 µg) from *P. brasiliensis* hyphal (H) and yeast (Y) cells using anti-rPbPga1 (5 µg/ml) and anti-pepI_PbPga1 (10 µg/ml). A single 88 kDa polypeptide (arrow) was detected. Donkey IgG anti-IgY conjugated to HRP (1∶20000) was used as the secondary antibody.

A polyclonal antibody anti-rPbPga1 was produced in chickens and the slot blot analysis revealed that this antibody was able to detect rPbPga1 over a wide range of concentrations *in* a dose dependent manner (Figure 3A). The specificity of the antibody was determined by western blotting against rPbPga1. The blot revealed that anti-rPbPga1 reacted with a single polypeptide of 74 kDa which is consistent with the molecular weight of the recombinant protein, rPbPga1. Western blots of total protein extracts from *P. brasiliensis* yeast and hyphal cells showed that anti-rPbPga1 reacted with a single polypeptide (88 kDa). Further, the anti-rPbPga1 reacted more strongly with the yeast polypeptide (Figure 3B). This finding agrees with the results from real time PCR in which this protein is up-regulated in yeast cells (data not shown). Furthermore, we produced another antibody against the non-glycosylated region of PbPga1 (Figure 1C), the anti-PepI_PbPga1 showed similar results to anti-rPbPga1 (Figure 3B). Pre-immune IgY did not react with total protein extracts from *P. brasiliensis* yeast or hyphal cells (data not shown).

### PbPga1 is localized on the surface of *P. brasiliensis* yeast

**Figure 4:**
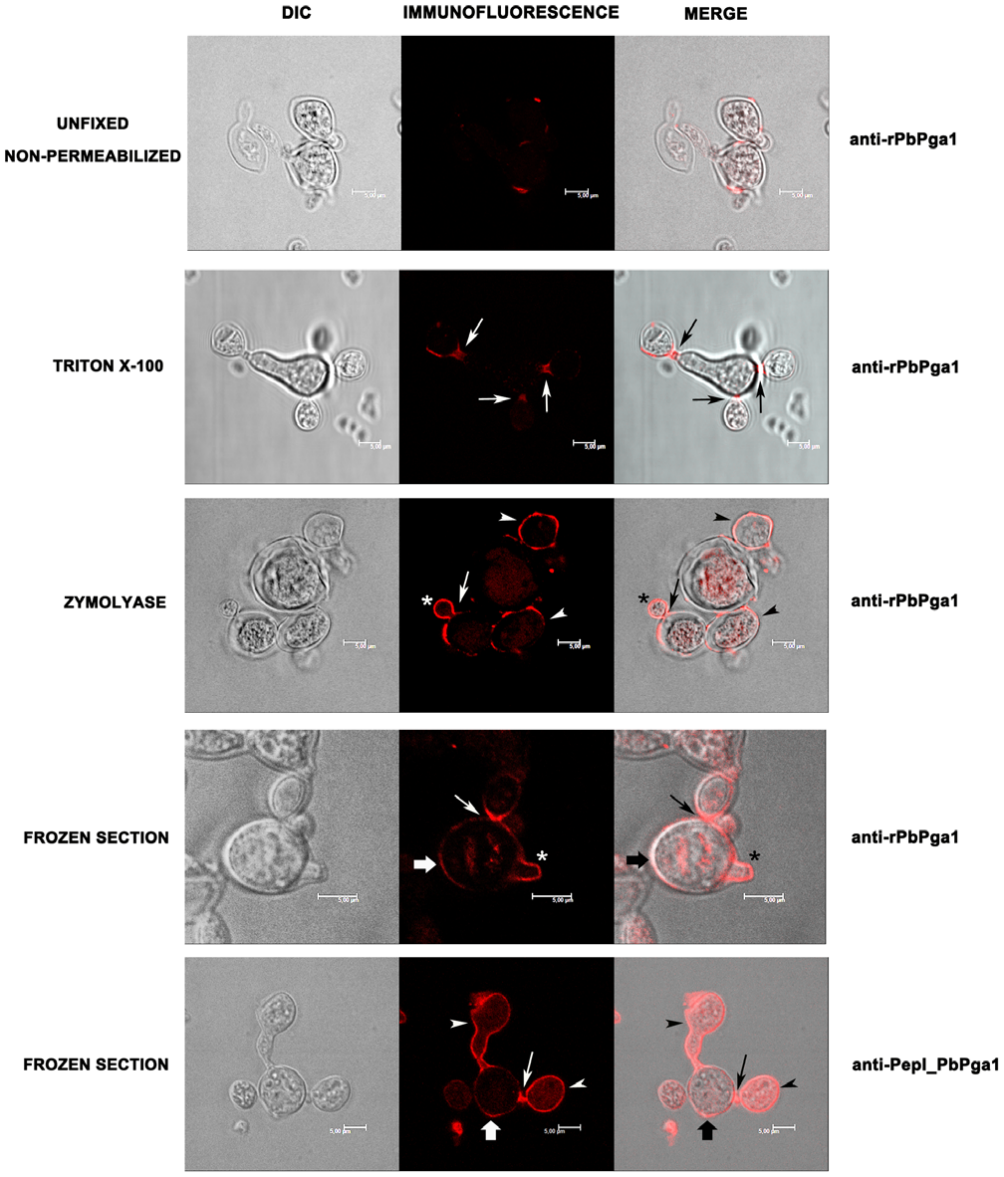
Localization of PbPga1 in *P. brasiliensis* yeast cells. Yeast cells were incubated with anti-rPbPga1. PbPga1 has a heterogeneous distribution on the cell surface of unfixed, non permeabilized yeast cells. Yeast cells fixed and permeabilized treated with Triton X-100 showed a heterogeneous distribution on the cell surface with stronger labeling at the bud necks (arrows). After enzymatic treatment with zymolyase PbPga1 was localized at the bud necks (arrows) and on the buds themselves (arrowheads). A strong staining is observed in regions that appear to be forming buds (asterisk). Fixed frozen sections of yeast cells were incubated with either anti-rPbPga1 or anti-pepI_PbPga1. The mother cells have a moderate homogeneous labeling on their surface (large arrows). There is a strong staining in the bud necks (arrows) and in growing buds (arrowheads). Labeling can also be observed in the cytoplasm with both anti-rPbPga1or anti-PepI_PbPga1. Donkey IgG anti-IgY conjugated to dylight 594 (1∶1000) was used as the secondary antibody.

In order to confirm the localization of PbPga1 on the surface of yeast cells, unfixed, non permeabilized yeast cells were labeled with anti-rPbPga1. PbPga1 was localized in a punctuate manner on the yeast cell surface, possibly at the septum between mother cells and buds (“bud necks”) (Figure 4). In order to elucidate the distribution of PbPga1 on the cell surface, fixed yeast cells were permeabilized with Triton X-100 or treated with zymolyase before immunostaining. The results showed that even after permeabilization or lysis of the cell wall, the labeling remained weak and punctuate in the mother cells and was stronger in the buds with a possible localization in regions that appear to be forming buds (Figure 4). To further characterize the localization of PbPga1, yeast cells were fixed with formaldehyde, and frozen sections were immunostained with anti-PbPga1 or with anti-pepI_PbPga1. The sections of yeast cells showed a moderate homogeneous labeling of the surface of the mother cells. An intense staining of the bud surface and bud necks was also observed. Furthermore, labeling was observed in the cytoplasm of the mother cells and buds (Figure 4).

### PbPga1 is O-glycosylated

**Figure 5:**
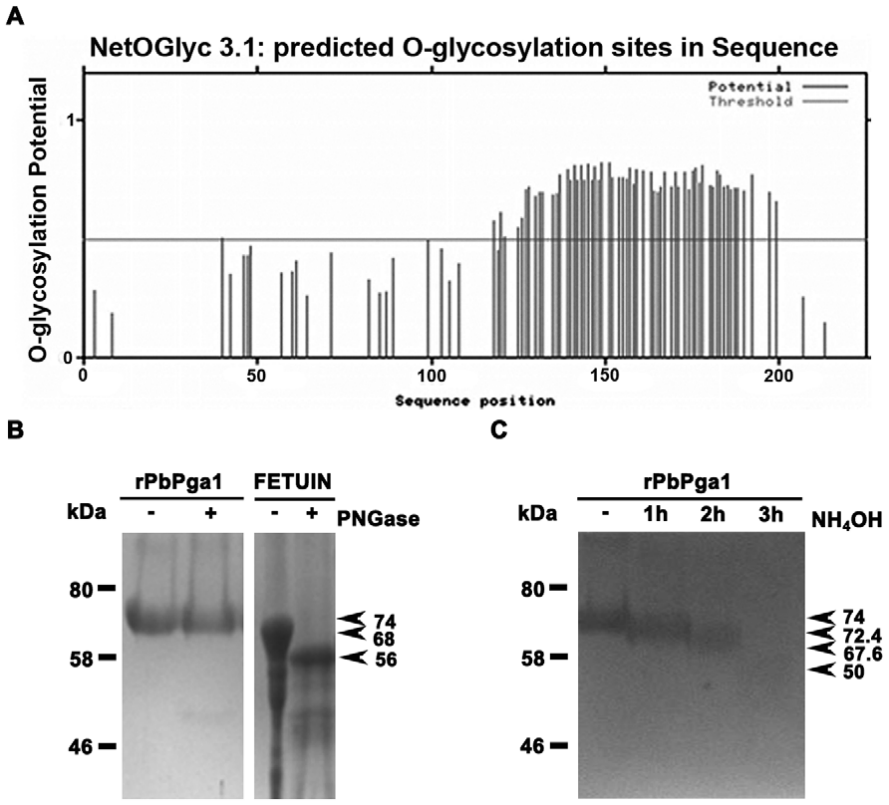
*P. brasiliensis* PbPga1 is *O*-glycosylated. (A) Several *O*-glycosylation sites were predicted in PbPga1 C-terminus by the NetOGlyc program. (B) Comassie stained SDS-PAGE gel of purified rPbPga1 and fetuin (control) treated with PNGaseF (PNGase) . (C) Comassie stained SDS-PAGE gel of purified rPbPga1 subjected to alkaline β-elimination.

Western blot analysis of both the protein cell extracts from *P. brasiliensis* yeast and hyphal cells and the culture supernatant of *P. pastoris* cells expressing rPbPga1 (Figures 3B and 2B, respectively) revealed bands whose apparent molecular masses were higher than the predicted size of PbPga1 (∼27 KDa). This observation could be explained by post-translational modifications in PbPga1, such as glycosylation. *In silico* analysis of PbPga1 sequence by NetOGlyc and NetNGlyc programs identified several potential *O*-glycosylation sites (Figure 5A), whereas no *N*-glycosylation sites were identified (data not shown). Consistently with the predicted absence of *N*-glycans linked to the protein, the molecular mass of rPbPga1 was maintained after PNGaseF digestion (Figure 5B). Nevertheless, the presence of *O*-glycans in PbPga1 was determined by alkaline β-elimination under ammonium hydroxide treatment (Figure 5C). The time-course effect of ammonium hydroxide exposure revealed a progressive reduction of rPbPga1 molecular mass from 74 to 50 kDa.

### rPbPga1 induces TNF-α and NO release by macrophages

**Figure 6:**
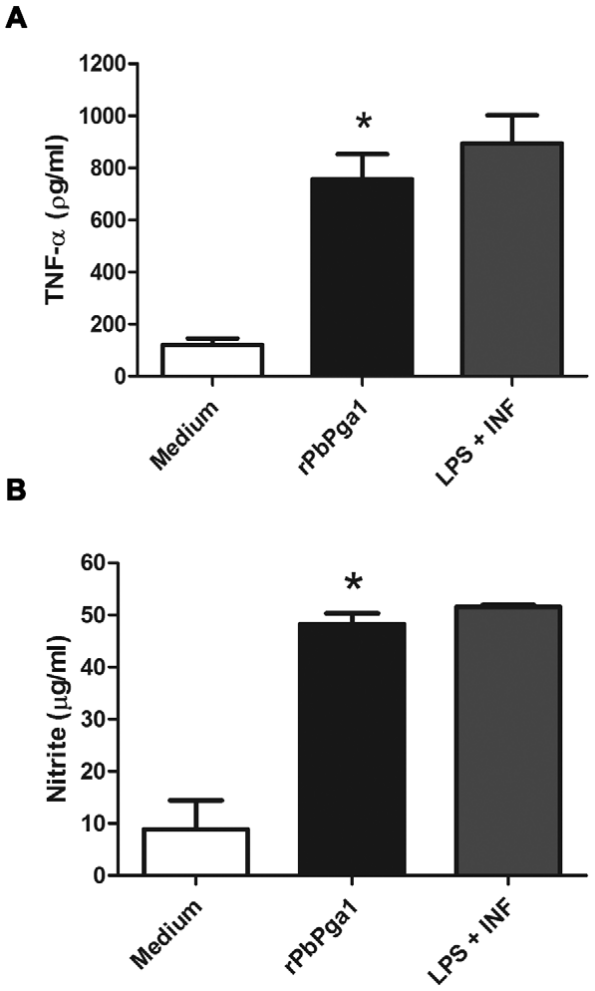
rPbPga1 activates macrophages. (A) Release of TNF-α and (B) production of NO by elicited resident murine macrophages following *in vitro* stimulation with rPbPga1. Cells were incubated with rPbPga1 (0.25 µg/ml) or LPS (10 µg/ml) and Interferon gamma (1.5 ng/ml), or culture medium for 48 h. Data is from a minimum of 3 independent experiments ± SD. * p<0,05 between rPbPga1 and medium.

We aimed to determine whether rPbPga1 could elicit cytokine release and/or nitric oxide (NO) production by incubation of mouse peritoneal macrophages with rPbPga1 for 48 h, since inflammatory mediators released by activated macrophages play a major role in the host resistance to *P. brasiliensis* infection. This stimulus induced the production of TNF-α (756.6±95.6 pg/ml) and NO (48.2±2.1 µg/ml) (Figure 6). There was a statistically significant difference (P<0.05) between rPbPga1 stimulus and medium in both experiments. On the other hand, rPbPga1 did not stimulate production of either IL-10 or IL-12 (data not shown). Together, these results demonstrated that rPbPga1 induces macrophages to produce important inflammatory mediators in the innate immune response against *P. brasiliensis* infection.

### rPbPga1 is recognized by sera from PCM patients

**Figure 7:**
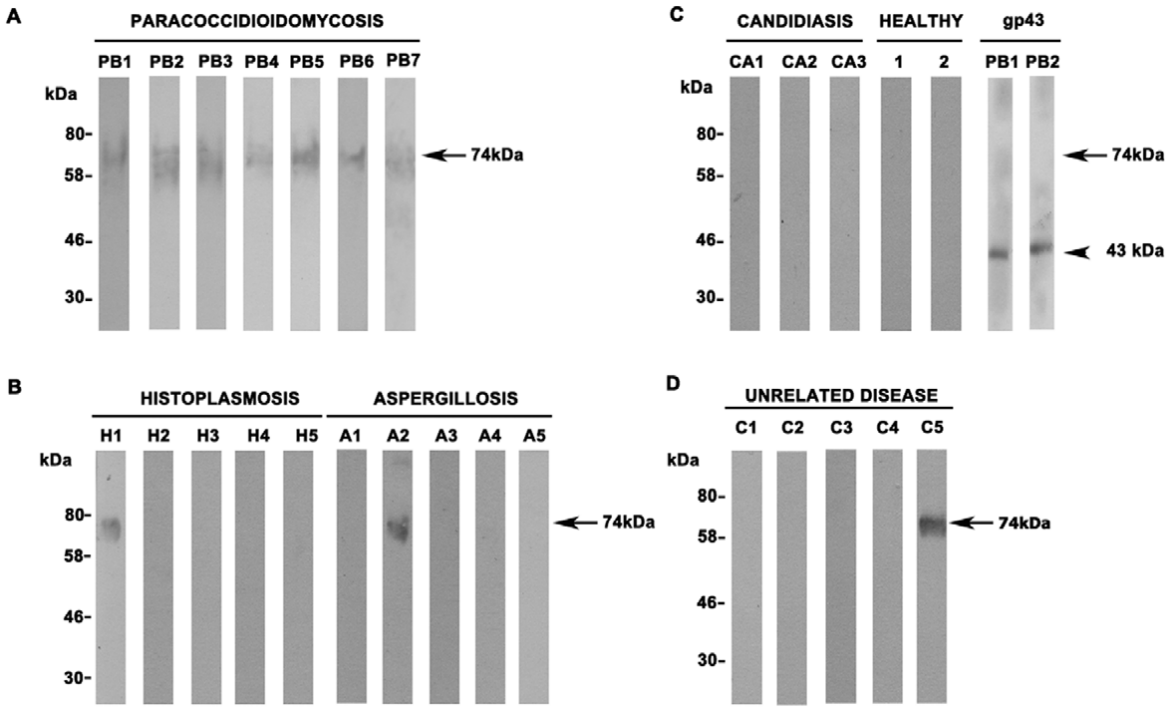
rPbPga1 is recognized by sera from PCM patients. (A) Purified rPbPga1 (1 µg) was subjected to SDS-PAGE followed by western blot analysis using sera from seven PCM patients (Pb1-Pb7) and a single 74 kDa polypeptide (arrow) was detected. (B) One histoplasmosis patient (H1) and aspergillosis patient (A2) reacted with rPbPga1 (arrow). (C) None of the three sera from candidiasis patients detected rPbPga1 (Ca1, Ca2 and Ca3). Sera from two healthy people were used as negative controls (1 and 2). Native *P. brasiliensis* Gp43 of 43 kDa (1 µg) was used as positive control (arrowhead). (D) Sera from patients with unrelated diseases were tested and one serum from a patient with pneumonia presented cross reactivity with rPbPga1 (C5). All primary antibodies were diluted to 1∶1000. Goat anti- human IgG was used as the secondary antibody.

It was of interest to determine if sera from PCM patients could recognize rPbPga1. Futhermore, we wanted to know if sera from patients with other mycosis such as histoplasmosis, aspergillosis and candidiasis would cross-react with rPbPga1. Western blot analysis showed that rPbPga1 was recognized by sera from 7 out of 7 PCM patients, 1 out of 5 histoplasmosis patients ,1 out of 1 aspergillosis patients , but not by any sera from the 3 candidiasis patients. As a positive control, the major antigen of *P. brasiliensis*, gp43 was tested against sera from two PCM patients. Sera from 2 healthy individuals and sera from 5 patients with unrelated diseases were used as negative controls. One serum from a patient with pneumonia presented cross reactivity with rPbPga1 (Figure 7). These results indicate that *P. brasiliensis* PbPga1 elicits the production of specific antibodies during PCM infection. In addition, a low rate of cross-reactivity was revealed by rPbPga1 incubation with sera from patients affected by other systemic mycosis.

## Discussion

This study identifies and characterizes PbPga1, a novel predicted GPI-anchored protein localized on the cell surface of *P. brasiliensis* yeast cells and up-regulated in the yeast pathogenic phase. The rPbPga1 is heavily glycosylated and it is able to activate cytokine release from peritoneal macrophages. Moreover, rPbPga1 is recognized by sera from PCM patients.

The search for expressed GPI-proteins in *Paracoccidioides* genomes revealed an ORF that codes for a protein with homology to predicted GPI-proteins that are restricted to members of the class Eurotiomycetes. The analysis of PADG_02460 genomic sequence showed a misprediction in the C-terminal sequence in the Broad Institute database. We have also identified a substantial number of other predicted GPI-proteins from *Paracoccidioides* strains with prediction errors in the automated annotated genome (Basso et col., personal communication). The newly annotated PADG_02460 (referred to as *PbPga1*) codes for a protein of 225 amino acids with close orthologs in *P. brasiliensis* strain Pb03 and *P. lutzii* strain Pb01. The *in silico* analysis of PbPga1 and its orthologs revealed that it contains all conserved domains found in Ser-Thr rich GPI-anchored proteins. The PbPga1 has no other paralog in *P. brasiliensis* Pb18 and its orthologs in other fungi are restricted to Eurotiales and Onygenales orders. To date, the only GPI-protein characterized from a mycelial fungus is *cspA* from *A. fumigatus* [27]. The restricted distribution of PbPga1 orthologs indicates that this protein may have specific roles in biological and/or pathological processes among these fungi.

In fungi, many transcriptional programs are triggered during morphological transition associated with pathogenicity [33], [34]. Thus, the differential expression of *PbPga1* in the yeast pathogenic form suggests a role for this protein during the morphological transition and PCM infectious process.

The present study shows that PbPga1 is distributed on the surface of *P. brasiliensis* yeast cells and shows a strong staining on the bud surface and at the bud necks. We hypothesize that due to its localization PbPga1 may be involved in cell wall morphogenesis. Interestingly, a similar pattern of localization was reported for a GFP tagged Chitinase (ChiaA) of *A. nidulans* [21], which is involved in cell wall remodeling during conidial and hyphal growth.

The large difference between the molecular mass predicted for PbPga1 (27 kDa) and the molecular mass revealed by Western blotting of protein extracts of *P. brasiliensis* cells using specific antibodies against rPbPga1 (88 kDa) is consistent with heavy *O*-glycosylation of the predicted Ser-Thr rich domain of the protein [35]. Indeed, this region serves as a spacer domain that helps to extend the N-terminal functional domain [36]–[38]. *O*-glycan modulates the function of secreted proteins by enhancing their stability and solubility, protecting them from protease activity, and acting as a sorting determinant for their delivery to yeast cell surface [39], [40]. Therefore, PbPga1 was expressed in *P. pastoris*, which is able to correctly glycosylate fungal recombinant proteins [41]–[46] and recombinant rPbPga1 expressed in *P. pastoris* was indicated by the detection of a protein with a molecular mass of 74 kDa, which is higher than the predicted molecular mass of PbPga1 (27 kDa) and in accordance with the molecular mass of the endogenous PbPga1 (88 kDa). The fact that *O*-glycosylation did occur in rPbPga1 was demonstrated by a β-elimination procedure, which resulted in a decrease in the molecular mass of the protein that can be attributed to the substitution of *O*-glycans by the NH^3+^ group from ammonium hydroxide [47].

It is well established that innate immunity constitutes a first line of protection against infectious propagules of *P. brasiliensis* [48]. Resident alveolar macrophage activation is trigged by interaction with the pathogen, and is followed by internalization and death of the microorganism through reactive oxygen species and lytic enzymes [5]. In this process, the cytokines and NO production by macrophages play a critical role which has been verified experimentally [49]–[56] and in human PCM [57]–[62]. In the present study, the ability of rPbPga1 to induce macrophages to produce TNF-α and NO was demonstrated. TNF-α favors the formation of organized granulomas, restricting fungal dissemination [63]. The NO production by activated macrophages at the initial stages of disease accounts for the cell fungicidal activity [55], [56]. Therefore, rPbPga1 may activate the innate immune response during *P. brasiliensis* infection.

In this study, we observed that sera from seven PCM patients were able to recognize purified rPbPga1. Furthermore, one serum from a histoplasmosis patient, one serum from an aspergillosis patient and one serum from a pneumonia patient, presented cross reactivity to rPbPga1. However, PbPga1 was not recognized by sera of candidiasis patients and sera from individuals with unrelated diseases or healthy individuals. Interestingly, *H. capsulatum* and *Aspergillus* sp are close relatives of *P. brasiliensis* and members of the Onygenales order. rPbPga1 hyperglycosylation may be the cause of this reactivity. The cross reactivity of glycoproteins with sera from other systemic mycoses has been recognized in other studies. For example, it has been shown that gp43 protein, a diagnostic marker for PCM, cross reacts with sera from individuals with other systemic mycosis probably due to its sugar epitopes [64].

Altogether, our results show that PbPga1 is an unreported predicted GPI-protein that is conserved exclusively in Eurotiomycetes. PbPga1 possess signals for GPI anchoring, for insertion into ER, a high number of predicted *O*-glycosylated residues and localizes at the surface of yeast cells. These characteristics are consistent with a role in cell wall organization. Additional biochemical assays are necessary to determine whether PbPga1 is transferred to the cell wall or is maintained as a plasma membrane protein. In addition, rPbPga1 induces the production of TNF-α and NO, macrophage mediators of the innate immune response to PCM. Moreover, rPbPga1 is recognized by sera from PCM patients, suggesting it induces humoral immunity during *P. brasiliensis* infection.

## Materials and Methods

### Strains and media

Virulent *P. brasiliensis* strain 18 (Pb18) was provided by Dr. Gustavo H. Goldman (University of São Paulo, Brazil). *P. brasiliensis* cells were grown at 35°C for the yeast form and 25°C for the hyphal form in modified YPD medium (0.5% casein peptone, 0.5% yeast extract, 1.5% glucose, pH 6.3). *Pichia pastoris* strain GS115 from the Multi-copy *Pichia* Expression Kit (Invitrogen Life Technologies, Inc., Grand Island, NY) was cultured at 30°C in (i) YPD medium (2% peptone, 1% yeast extract, 2% glucose, 2% agar, pH 6.5), (ii) minimal medium (1,34% yeast nitrogen base-YNB, 4×10^−5^% d-biotin) containing either 2% dextrose or 0.5% methanol, (iii) K-BMGY medium (1% yeast extract, 2% tryptone, 100 mM potassium phosphate, pH 6.0, 1.34% YNB, 4×10^−5^% biotin, 1% glycerol, 0.4 M KOAc) or (iv) S-BMMY induction medium (1% yeast extract, 2% tryptone, 100 mM potassium phosphate, pH 6.0, 1.34% YNB, 4×10^−5^% biotin, 0.5% methanol, 0.2 M Sorbitol). Plasmids were amplified in *Escherichia coli* (*E. coli*) in Luria-Bertani medium (1% tryptone, 0.5% yeast extract, 1% NaCl, pH 7.0) with 100 µg ampicillin per ml. Solid media were obtained by adding 1.5% agar.

### Ethics Statement

All serum samples were collected in accordance with the ethical guidelines and written consent protocols mandated by the review board of Hospital das Clínicas, School of Medicine of Ribeirão Preto, University of São Paulo (protocol # 13982/2005).

### Identification of PbPga1 in *P. brasiliensis* strain Pb18

Expressed Sequence Tags (ESTs) from *P. brasiliensis* strain 18 (dos Reis Marques *et al.*, 2003) were used as queries for a tBLASTp search into the genome of *P. brasiliensis* strains (Pb03 and Pb18) and *P. lutzii* strain (Pb01) through the Fungal Genome Initiative of the Broad Institute (http://www.broadinstitute.org/scientific-community/science/projects/fungal-genome-initiative/fungal-genome-initiative). The retrieved open reading frame (ORF) sequences were analyzed for GPI-anchoring signals, as described in the next section. Among the predicted GPI-anchored proteins, the ORFs PABG_00068 and PADG_02460 from *P. brasiliensis* strains (Pb03 and Pb18, respectively) and the ORF PAAG_04708 from *P. lutzii* strain (Pb01) were manually inspected for predicted introns/exons by the Augustus program using different fungi organisms (http://augustus.gobics.de/).

### In silico analysis of *P. brasiliensis* PbPga1

The 225 amino acid sequence of *P. brasiliensis* PbPga1 (GenBank: JN967755) was used as the query sequence for PSI-BLAST searches against non-redundant databases at NCBI (http://blast.ncbi.nlm.nih.gov/Blast.cgi) and fungi databases available at Broad Institute. We identified potential PbPga1 orthologs based on amino-acid identity (≥30%) and global pairwise alignment (≥60%) to PbPga1 sequence. The amino acid sequence with the highest identity to PbPga1 in each fungus was considered its ortholog and selected for further analysis. The sequences were submitted to analysis of (i) signal peptide cleavage site at N-terminus using the SignalP3.0 program (http://www.cbs.dtu.dk/services/SignalP/), transmembrane helices by TMHMM program (http://www.cbs.dtu.dk/services/TMHMM/) and (iii) GPI anchoring sites at C-terminus using the GPI-SOM program (http://gpi.unibe.ch/) and the Fraganchor program (http://navet.ics.hawaii.edu/~fraganchor/NNHMM/NNHMM.html). Multiple sequence alignment and the percentage of amino-acid identity were calculated using the CLUSTALW2 program (http://www.ebi.ac.uk/Tools/msa/clustalw2/). Analysis of predicted molecular weight and serine/threonine content was carried out using the Bioedit sequence alignment editor program.

### Real Time quantitative PCR analysis

Total RNA was extracted from yeast and hyphal cells of *P. brasiliensis* using Trizol reagent (Invitrogen Life Technologies, Inc.) according to the manufacturer's specifications. Total RNA was resuspended with DEPC treated water, quantified by spectrophotometry and stored at −80°C. Genomic DNA was removed from total RNA samples by DNAseI (New England Biolabs, Ipswich, MA) digestion following the manufacturer's instructions. Total RNA was reversibly transcribed to cDNA using Improm-II reverse transcriptase (Promega Corporation, Madison, WI) according to manufacturer's specifications. qRT-PCR was carried out using the Kit SYBR Green PCR master mix (Applied Biosystems, Carlsbad, CA) using the *PbPga1* specific primers PbPga1_RT_F (5′ GAACCGACCTCCCCAACAAT 3′) and PbPga1_RT_R (5′ GTTGATGTTACGGGACCTTCCA 3′). Relative amounts of total cDNA were calculated by *L34* normalization using the specific primers L34_RT_F (5′ AAGAAAGGAACCGCACCAAA 3′ ) and L34_RT_R (5′ TCGGAGGGCAGGAATGC 3′). Reactions were performed at 50°C for 2 min, 95°C for 5 min and 40 cycles (95°C, 15 sec; 60°C, 1 min). The concentration of each primer pair and the amount of cDNA used in qRT-PCR were empirically determined (5 pmols and 1∶100 dilution, respectively). The expression levels of the predicted genes were calculated using the ΔCt (Cycle threshold) method and normalized to *L34* gene expression. Reaction specificity was confirmed by dissociation curve analysis (95°C, 15 min; 60°C, 20 sec; 95°C, 15 sec). Results are expressed as the mean of duplicate measurements in 3 independent experiments. Negative controls were included with each round of reactions to confirm that there was no contamination with genomic DNA by using a RNA sample without conversion to cDNA.

### PCR amplification of genomic DNA and cDNA

*P. brasilensis* genomic DNA (0.8 kb) and total cDNA were used as templates to amplify by polymerase chain reaction (PCR) a fragment of 0.6 kb (position 1 to 678) of *PbPga1* ORF. PCR was carried out using Taq DNA polymerase (New England Biolabs) according to manufacturer's specifications. PCR reactions were performed at 94°C for 5 min followed by 29 cycles (94°C, 2 min; 54°C, 40 sec; 68°C, 1 min) and a final step at 54°C for 7 min. The primer BamHI_PbPga1-F (5′ CGGGATCCCCATGCACTCAATCATCTACTTC 3′) and the primer HindIII_PbPga1-R2 (5′ CGAAGCTTCTACAGGAAAGCTATCAGTCCC 3′) were used at final concentration 10 pg and the template concentration was 10 ng per reaction. Negative controls were included with each round of reactions to confirm that there was no contamination with RNA or genomic DNA. PCR samples were analyzed by electrophoresis in 1% agarose gel after staining with GelRed (Biotium, Inc. Hayward, CA). The expected PCR fragments were 694 bp for cDNA as template and 894 bp for genomic DNA as template.

### rPbPga1 expression in *P. pastoris*

A synthetic *PbPga1* ORF sequence was designed with optimized *P. pastoris* codons (http://www.kazusa.or.jp/codon). The recombinant 587 bp *PbPga1* (*rPbPga1*) coding sequence was synthesized by a commercial vendor (GenScript USA Inc. Piscataway, NJ) and the accuracy of the DNA synthesis was verified by DNA sequencing. The *rPbPga1* coding sequence lacks the native secretion signal at N-terminus and the GPI-anchoring signal at C-terminus. The synthetic *rPbPga1* has a *EcoR*I restriction site at 5′ end, an in-frame His-tag coding sequence upstream of the start codon and two stop codons (TAATAA) downstream. The last codon is followed by an *Not*I restriction site. The *rPbPga1* was ligated into the vector pPIC9K at the *EcoR*I and *Not*I restriction sites yielding the recombinant vector pPIC9K-rPbPga1. The recombinant vector pPIC9K-rPbPga1 (*Pp*CV1) and the empty vector pPIC9K were linearized by *Sal*I digestion and transformed into the *P. pastoris* strain GS115 (His^−^ ; *Mut* ^+^) using the electroporation method described for the Multi-copy *Pichia*Expression Kit (Invitrogen Life Technologies, Inc.). The transformants were selected on YPD medium containing G418 (2 mg/ml) (Amresco LLC, Solon, OH). *Mut* ^+^ and *Mut^s^* phenotypes of the transformants were evaluated by growth on minimal medium agar containing either 2% dextrose or 0.5% methanol as the primary carbon source. The correct clone insertion was verified by PCR using genomic DNA from *P. pastoris* transformants using the primer 5′AOX1 (5′ GACTGGTTCCAATTGACAAGC 3′) and the primer 3′AOX1 (5′ GCAAATGGCATTCTGACATCC 3′) followed by 1% agarose gel electrophoresis. *P. pastoris Pp*CV1 or pPIC9K transformed cells were cultivated in YPD medium at 30°C for 16–18 h. Culture aliquots (100 µl) were transferred to 50 ml of K-BMGY medium and grown under agitation at 30°C. Log-phase cells were harvested by centrifugation (2,000× *g*, 5 min) and then resuspended to OD_600 = _1.0 in S-BMMY induction medium. The cells were cultured at 18°C and rPbPga1 expression was induced by adding methanol (0.5% final concentration) at 24 h intervals. After 96 h of induction, culture supernatants were collected and 10 µl aliquots were analyzed by 10% SDS-PAGE electrophoresis (Laemmli,1970) followed by silver staining [65].

### rPbPga1 purification

The recombinant rPbPga1 was purified by one-step purification His-Bind affinity chromatography (Qiagen Inc. Valencia, CA). The fermentation culture (100 ml) was centrifuged (10,000× *g*, 15 min, 4°C), the cell-free supernatant was dialyzed against 1× PBS. The dialyzed sample was filtered and concentrated to 5 ml by using a 30 kDa cut-off Amicon filter (© EMD Millipore Corporation, Billerica, MA, USA). The supernatant was incubated for 1 hour at 4°C on a Ni-NTA column (Qiagen). The unbound proteins were washed out with buffer A (1× PBS, pH 7.8; 20 mM imidazole). The rPbPga1 was eluted with buffer B (1× PBS, pH 7.8; 200 mM imidazole). The fractions were dialyzed against 1× PBS and the concentrated samples were analyzed by 10% SDS-PAGE followed by silver staining. The protein concentration was determined by BCA assay (MicroBCA Protein Assay Reagent Kit, Thermo Fisher Scientific Inc. Rockford, IL) using BSA as a standard. Samples were tested for LPS (Lipopolysaccharide) contamination using the Limunus Amebocyte Lysate QCL-1000 (Lonza Inc. Allendale, NJ).

### Mass spectrometry

The identity of rPbPga1 was confirmed by mass spectrometry. Band corresponding to rPbPga1 were cut from polyacrylamide gels and treated with trypsin for 18 hours at 37°C. The peptides were extracted from the gels using 60% acetonitrile in 0.2% trifluoroacetic acid (TFA), concentrated by vacuum and desalted using C18 reverse phase micro-columns (OMIX Pipete tips, Varian Medical Systems, Inc. Palo Alto, CA). Peptides were eluted from the micro-columns into the mass spectrometer sample plate with 3 µl of matrix solution (α-cyano-4-hydroxycinnamic acid in 60% aqueous acetonitrile containing 0.2% TFA). Mass spectra of the digestion products were acquired in a 4800 MALDI-TOF/TOF instrument (Applied Biosystems) in reflector mode and were externally calibrated using a mixture of peptide standards (Applied Biosystems). Collision-induced dissociation MS/MS experiments of selected peptides were performed. Proteins were identified by local database searching with peptide m/z values using the MASCOT program, with the following search parameters: monoisotopic mass tolerance, 0.08 Da; fragment mass tolerance, 0.25 Da; methionine oxidation, as possible modification and one missed tryptic cleavage allowed.

### Production of chicken polyclonal antibodies

Five month-old egg-laying Rose Leghorn chickens were housed in individual cages with food and water *ad libitum*. At day zero, 80 µg of rPbPga1 or pepI_PbPga1 (peptide from the non-glycosylated region of PbPga1: PVNLFLQS) conjugated to *Keyhole Limpet Hemocyanin* (*KLH*) (Gen Script), were resuspended in PBS and emulsified with an equal volume of complete Freund's adjuvant (Sigma-Aldrich Inc. St. Louis, MO).The mixture was injected at two sites in the intrapectoralis muscle. Booster injections of 80 µg of antigen in incomplete Freund's adjuvant (Sigma-Aldrich) were administered three times (on days 14, 28 and 32). Starting at day 45, the eggs were collected daily and stored at 4°C until antibody purification.

### IgY extraction from Egg Yolk

IgY was purified from fat-free egg yolk using ammonium sulfate precipitation (Schwarzkopf and Thiele 1996). The yolk was separated from the egg white, diluted 9× with MilliQ water (Millipore) and was stirred continually for 6 hours. The yolk samples were centrifuged at 11,000×*g* for 30 min at 4°C, to separate the lipids. The supernatant containing IgY was filtered through sterile gauze and IgY was precipitated by adding a saturated solution ammonium sulfate to a concentration of 45% (v/v) at 4°C with continuous stirring for 16–18 h. The precipitate was collected by centrifugation at 11,000×*g* for 30 min at 4°C. The pellet was resuspended to the initial volume and the precipitation step was repeated twice. The IgY pellet was resuspended in 2 mL MilliQ water (Millipore) and dialyzed against PBS. The concentration of IgY was estimated by spectrophotometrically at an absorbance of 280 nm.

### Titration of IgY anti-PbPga1

Different concentrations (0.1, 0.5, 1 and 5 µg) of rPbPga1 were immobilized on nitrocellulose membranes (Hybond-P, GE Healthcare Amersham, Pittsburgh, PA). Nonspecific binding was blocked by incubation with 5% skim milk in TBS-T (150 mM NaCl, 50 mM Tris, 0.05% Tween) for 1 hour, at room temperature (RT). After incubation, the membranes were washed five times for 5 min with TBS-T at RT. The rPbPga1 was detected by incubating the membranes with IgY anti-rPbPga1 or IgY anti-pepI_PbPga1 at various concentrations (1, 5 and 10 µg/mL) for 60 min at RT. Membranes were washed five times for 5 min with TBS-T at RT and incubated with donkey IgG anti-chicken conjugated to peroxidase (Jackson ImmunoResearch Laboratories, Inc. West Grove, PA) diluted 1∶20,000 in TBS-T for 45 min at RT. Controls were incubated with secondary antibody or preimmune IgY. The membranes were developed using an ECL chemoluminescence kit (GE Healthcare Amershan Inc.).

### Immunofluorescence

*P. brasiliensis* yeast cells were cultured for 2 days in YPD medium, washed in PBS and fixed in 2% formaldehyde (Electron Microscopy Sciences, Hatfield, PA) in PBS for 20 min at RT. After wash twice in PBS yeast cells were permeabilized or not with 20 µg/ml of zymolyase 20 T (Sigma-Aldrich) or Triton X-100 (1∶1000) diluted in PBS, for 45 min at RT. For some experiments yeast cells were embedded in O.C.T -Tissue Tek (Electron Microscopy Sciences).The samples were frozen in acetone cooled with dry ice and then stored at −20°C. Six-micron sections were obtained using a cryostat (Microm D6900 GmbH, Heidelberg, Germany) and placed on gelatinized glass slides.

For immunolabeling the samples were rinsed thoroughly in PBS and blocked with 2% BSA in PBS for one hour. The samples were incubated for 45 min at RT with IgY anti-PbPga1 (20 µg) or IgY anti-pepI_PbPga1 (20 µg) diluted in PBS. The samples were rinsed in PBS and incubated with secondary antibody donkey IgG anti-chicken IgY conjugated with Dylight 594 (R&D Systems, Minneapolis, MN) diluted 1∶1000 in PBS, and coverslips mounted with Fluoromount-G (Electron Microscopy Sciences) and examined with a Leica SP5 scanning confocal microscope (Leica Microsystems, Manheim, Germany). For controls the primary antibody was omitted or samples incubated with pre immune IgY. All controls were negative. Figures were prepared using Adobe Photoshop CS5 (Adobe Systems, Inc., San Jose, CA).

### Protein extraction

*P. brasiliensis* yeast and hyphal cells were grown under agitation and collected by centrifugation (12,000×*g* for 10 min). Cell pellets were frozen in liquid nitrogen and mechanically disrupted in a mortar until a fine powder formed. The broken cells were transferred to a glass tube containing calcium-Tris buffer (20 mM Tris-HCl pH 8.8, 2 mM CaCl_2_, 1 mM EDTA, 40 µg/ml pepstatin A, 40 µg/ml aprotinin, 20 µg/ml leupeptin, 4 mM PMSF (Roche Genentech Inc., San Francisco, CA). Homogenization was carried out by vortexing the sample for 30 min at 4°C with zirconium beads. The cell extract was centrifuged for 20 min at 12,000×*g* at 4°C and the supernatant was stored at −20°C.

### Western blotting

Total protein extracts from *P. brasiliensis* yeast and hyphal cell samples were submitted to 10% SDS-PAGE electrophoresis.and transferred to nitrocellulose membranes (Hybond-P, GE Healthcare Amersham) for two hours at 70V in transfer buffer (1.9% Tris-Base, 9.1% glycine). Membranes were stained in 0.5% Ponceau S in 0.3% trichloroacetic acid (TCA) and washed in 0.5% TCA. Nonspecific interactions were blocked by incubation with TBS-T +5% skim milk for 1 hour at RT. Membranes were washed 5 times for 5 min with TBS-T. The membranes were immunostained using IgY anti-PbPga1 (5 µg) or IgY anti-pepI_PbPga1 (10 µg). For immunoblotting the purified rPbPga1 (1 µg) or native gp43 (1 µg), membranes were immunostained using mouse monoclonal IgG anti-His (1∶1000) (Sigma-Aldrich) or human serum (1∶1000). For immunobloting with human serum, sera from 6 PCM patients, 5 histoplasmosis patients, 5 aspergillosis patients, 3 candidiasis patients, 5 sera from patients with unrelated diseases and 2 sera from healthy volunteers were used. Membranes were washed five times with TBS-T plus 5% skimmed milk for 5 min. Membranes were incubated with donkey anti-chicken IgG (1∶20000) (Jackson ImmunoResearch) or goat anti-human IgG (1∶10000) (Sigma-Aldrich) conjugated to horseradish peroxidase in TBS-T with 5% skim milk for 40 min. Immunoreactive proteins were detected using ECL (Amersham Biosciences) followed by exposure to X-ray film (Amersham Biosciences). Controls were incubated only with secondary antibodies.

### Glycosylation analysis

The programs NetNGlyc 1.0 (www.cbs.dtu.dk./services/NetNGlyc) and NetOGlyc 3.1(www.cbs.dtu.dk./services/NetOGlyc) were used for prediction of *N* and *O*-glycosylation sites, respectively. Enzymatic *N*- and *O*-deglycosylation assays were performed with the enzymatic Protein degylcosylation kit (Sigma-Aldrich) according to the manufacture's specifications using denatured bovine fetuin (DBF) as a control. Protein chemical *O*-deglycosylation was carried out by alkaline β-elimination using 25% ammonium hydroxide [47]. rPbPga1 (25 µg) was added to 200 µl of ammonium hydroxide and incubated at 45°C for different time points (1, 2 and 3 hours). Samples were withdrawn and the ammonium hydroxide was removed by Amicon Ultra 10 kDa cut-off filters (Millipore). Samples were submitted to 10% SDS-PAGE electrophoresis followed by Comassie blue staining.

### TNF-α release and Nitric Oxide production by perithoneal macrophages

C57BL/6 mice were intraperitoneally injected with 1 ml of sterile 3% sodium thioglycolate (Sigma Aldrich), as previously described [66]. After 3 days, the animals were sacrificed and the peritoneal lavage was collected by laparotomy with 5 ml of 1× PBS. Cells were stored on ice, washed in 1× PBS and suspended in RPMI 1640 medium (Flow Laboratories) containing 2 mM L-glutamine, 50 mM 2-mercaptoethanol, 100 units/ml penicillin, 100 µg/ml streptomycin (Sigma-Aldrich) and 5% heat-inactivated fetal calf serum (Life Technologies, Gibco) and aliquoted in wells of 24-well cell culture plates (2×10^6^ cells/well). Following incubation at 37°C by 40 min, non-adherent cells were removed by washing with RPMI 1640. The adherent cells were incubated with rPbPga1 (0.25 µg/ml) and cultured for 48 hours at 37°C in a humidified 5% CO_2_ atmosphere. The culture supernatants were harvested by centrifugation (5000× *g*, 10 min, 4°C) and stored at −20°C. TNF-α levels in the culture supernatants were quantified using an ELISA OptEIA Set mouse kit (Pharmigen), as previously described [66], [67]. Cytokine concentrations were determined using a standard curve of murine recombinant cytokines. The production of nitric oxide was quantified by measuring the accumulation of nitrite in the supernatants using the Griess reaction, as previously described [66], [67]. The results were statistically evaluated by analysis of variance one-way ANOVA, Tukey test, differences were considered statistically significant when p<0.05. The analysis was performed using the GraphPad Prism 5.

## Supporting Information

Figure S1 **DNA sequences of** ***PbPga1*** **, PADG_02460 (Pb18), PABG_00068 (Pb03) and PAAG_04708 (Pb01).** Predicted start codons and stop codons are represented by grey boxes and red boxes, respectively. Introns are limited by canonical GT/AG represented by black boxes.
(TIF)

Figure S2 ***PbPga1*** **orthologs in several fungi.** The CLUSTALW2 protein phylogenetic tree was generated based on fungal PbPga1 predicted sequences obtained from the Broad Institute database. The scale bar corresponds to 0.1 amino acid changes per site.
(TIF)

Figure S3 **Mapping of tryptic peptides obtained from rPbPga1.** The gel band was trypsin digested and analyzed by nanoelectrospray using MS scanning from 600 to 4000 amu in the positive ion mode for protonated peptide detection. Each ion present in the spectrum above was subjected to collision-induced dissociation to produce a fragment ion pattern.
(TIF)
