# Supplementary figures and images for: Characterization of PbPga1, an Antigenic GPI-Protein in the Pathogenic Fungus Paracoccidioides brasiliensis
Source: PLoS One. 2012 Sep 14;7(9):e44792. doi: 10.1371/journal.pone.0044792 (PMC3443090; doi:10.1371/journal.pone.0044792)

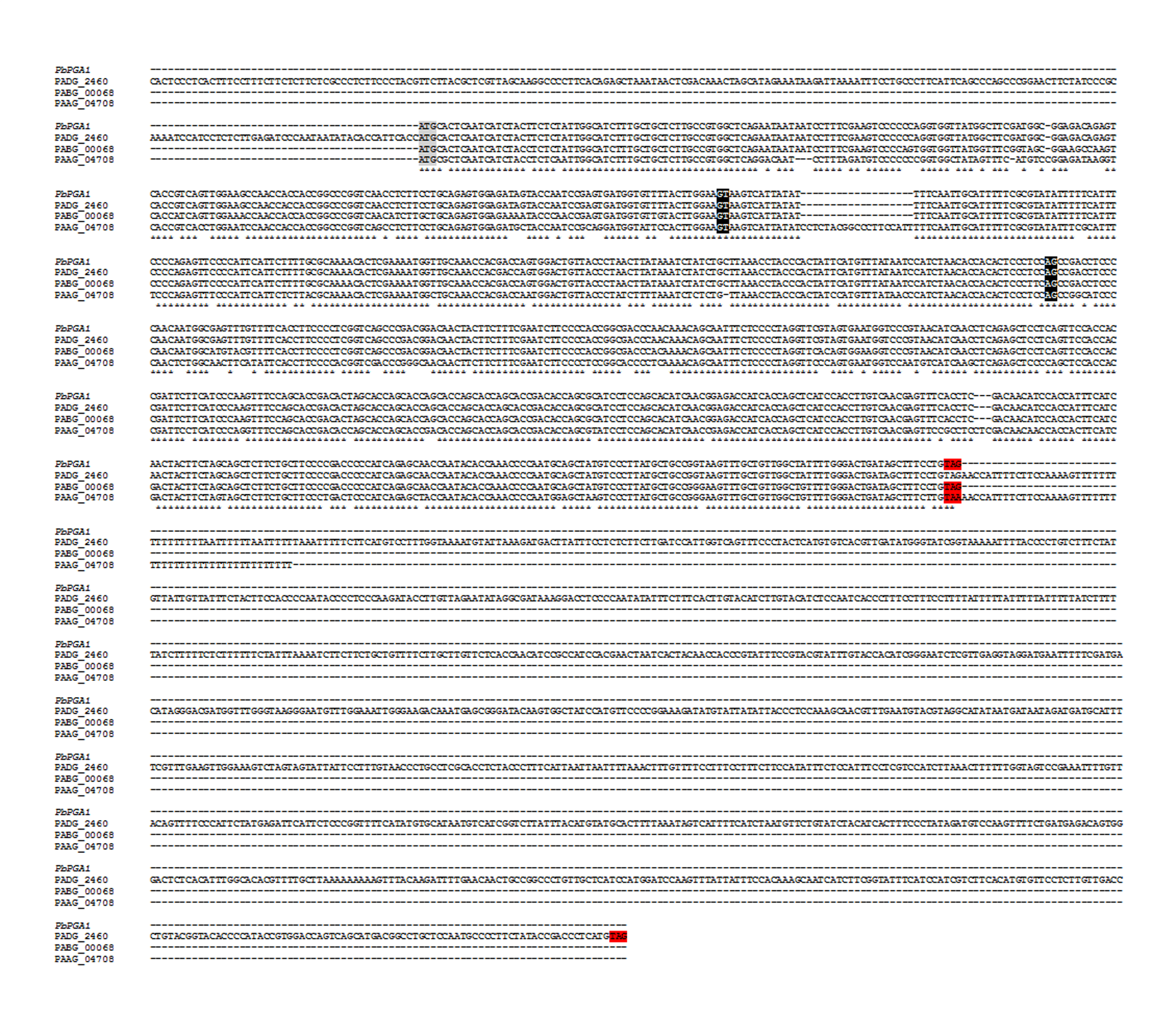

Supplement: Figure S1 — DNA sequences of PbPga1 , PADG_02460 (Pb18), PABG_00068 (Pb03) and PAAG_04708 (Pb01). Predicted start codons and stop codons are represented by grey boxes and red boxes, respectively. Introns are limited by canonical GT/AG represented by black boxes. (TIF) [file pone.0044792.s001.tif]

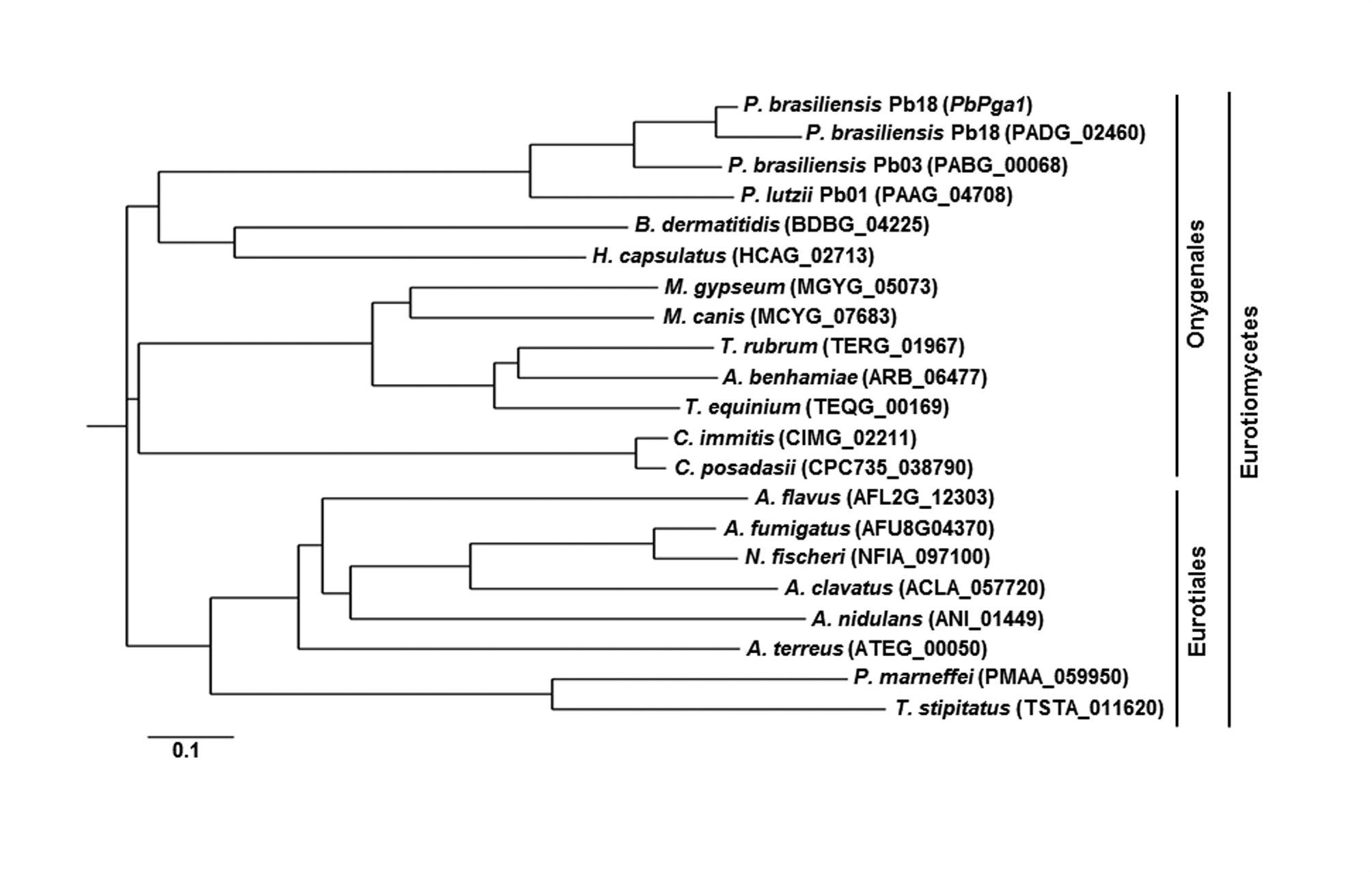

Supplement: Figure S2 — PbPga1 orthologs in several fungi. The CLUSTALW2 protein phylogenetic tree was generated based on fungal PbPga1 predicted sequences obtained from the Broad Institute database. The scale bar corresponds to 0.1 amino acid changes per site. (TIF) [file pone.0044792.s002.tif]

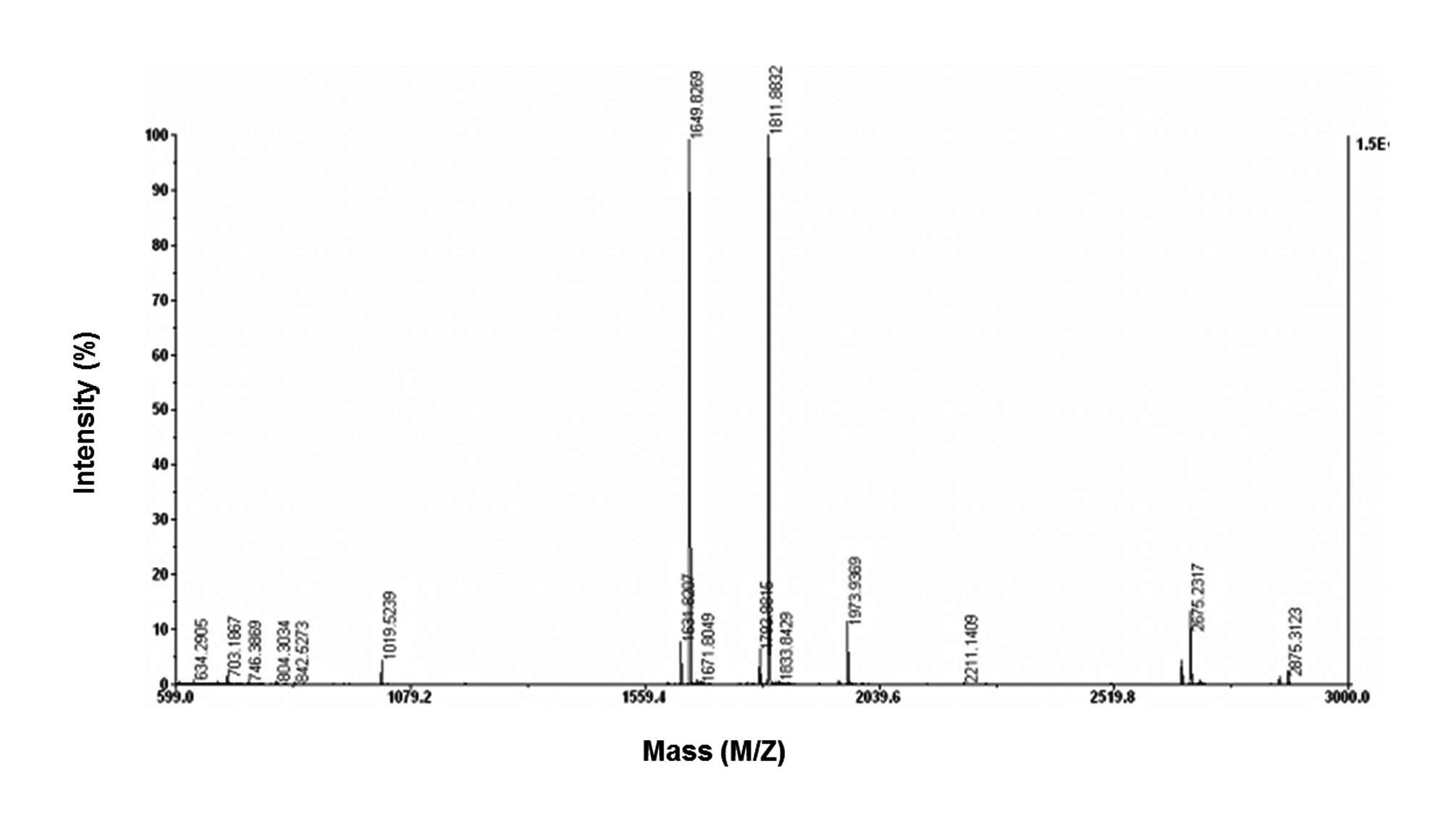

Supplement: Figure S3 — Mapping of tryptic peptides obtained from rPbPga1. The gel band was trypsin digested and analyzed by nanoelectrospray using MS scanning from 600 to 4000 amu in the positive ion mode for protonated peptide detection. Each ion present in the spectrum above was subjected to collision-induced dissociation to produce a fragment ion pattern. (TIF) [file pone.0044792.s003.tif]
